# Fluorescence-guided lymphadenectomy in gastric cancer surgery: a novel intraoperative indocyanine green injection

**DOI:** 10.1590/0102-672020260000031e1960

**Published:** 2026-09-28

**Authors:** Enrique LANZARINI, Enrique CRUZ, Pascale SALLABERRY, Deycies GAETE, Ignacio FERNÁNDEZ, Maher MUSLEH

**Affiliations:** 1 Hospital Clínico Universidad de Chile – Santiago, Metropolitan Region, Chile.; 2 Clínica Universidad de Los Andes – Santiago, Metropolitan Region, Chile.; 3 Clínica RedSalud Vitacura – Santiago, Metropolitan Region, Chile.

**Keywords:** Stomach Neoplasms, Lymphadenectomy, Indocyanine Green, Fluorescence, Gastrectomy, Neoplasias Gástricas, Linfadenectomia, Verde de Indocianina, Fluorescência, Gastrectomia

## Abstract

**Background:**

Fluorescence-guided lymphadenectomy using indocyanine green (ICG) improves intraoperative visualization of gastric lymphatic drainage during gastrectomy. Most reported techniques rely on preoperative endoscopic injection, and standardized intraoperative protocols are limited.

**Aims:**

To evaluate the feasibility, safety, and lymph node retrieval of intraoperative subserosal ICG injection for fluorescence-guided D2 lymphadenectomy during laparoscopic gastrectomy.

**Methods:**

A prospective descriptive pilot study was conducted at two institutions. Consecutive patients with gastric adenocarcinoma undergoing laparoscopic total or subtotal gastrectomy with curative intent and D2 lymphadenectomy were included. ICG was injected intraoperatively into the anterior gastric wall (two injections of 1.25 mg) after diagnostic exploration. Outcomes included feasibility, safety, fluorescence distribution across D2 stations, and total lymph node retrieval.

**Results:**

Twenty patients were included. The protocol was successfully implemented in all cases without technical difficulties or ICG-related adverse events. Complete D2 lymphadenectomy was achieved in 100% of patients. The median number of retrieved lymph nodes was 39 (range 29–73), with =30 nodes in 95% of cases. Fluorescent nodes were consistently identified in stations 3, 4, 6, and 7, with variable distribution in stations 5, 9, 8a, 11, 1, and 2, and absent in station 12a.

**Conclusions:**

Intraoperative subserosal ICG injection is a feasible, safe, and reproducible technique that enables consistent visualization of major gastric lymphatic basins without requiring preoperative endoscopic injection.

## ARTICLE HIGHLIGHTS

This study describes a novel intraoperative indocyanine green injection protocol for fluorescence-guided D2 lymphadenectomy during laparoscopic gastrectomy.

The protocol was feasible, safe, and reproducible in 20 consecutive patients undergoing curative-intent surgery for gastric cancer.

Intraoperative fluorescence enabled real-time visualization of major gastric lymphatic basins and systematic assessment of all D2 lymph node stations.

This approach avoids the need for preoperative endoscopic injection and may offer a practical framework for standardizing fluorescence-guided lymphadenectomy.

## Summary

**CENTRAL MESSAGE:**

Surgical resection with D2 lymphadenectomy continues to be the cornerstone of curative treatment of gastric cancer. However, the complex and variable vascular and lymphatic anatomy of the stomach makes lymph node dissection a technically demanding component of gastrectomy. Achieving an adequate lymphadenectomy is crucial not only for reducing residual disease, but also for ensuring accurate pathological staging, which directly influences prognosis and the indication for adjuvant therapy. Fluorescence-guided surgery with indocyanine green (ICG) has gained attention as a tool to improve intraoperative visualization of lymphatic drainage pathways. Because it allows real-time visualization of lymphatic flow and nodal stations under near-infrared imaging, this approach may improve the precision, reproducibility, and safety of lymphadenectomy.

**PERSPECTIVES:**

This study describes a novel intraoperative ICG injection protocol for fluorescence-guided D2 lymphadenectomy during laparoscopic gastrectomy, and demonstrates its feasibility, safety, and reproducibility in a consecutive cohort. The protocol enabled consistent real-time visualization of major gastric lymphatic basins, and allowed systematic assessment of fluorescence across all D2 lymph node stations without requiring preoperative endoscopic injection. These preliminary findings support the technical applicability of this approach, and should be further evaluated in larger series aimed at validating its clinical utility and exploring its potential role within standardized fluorescence-guided lymphadenectomy workflows.

## VISUAL ABSTRACT

**Figure:**
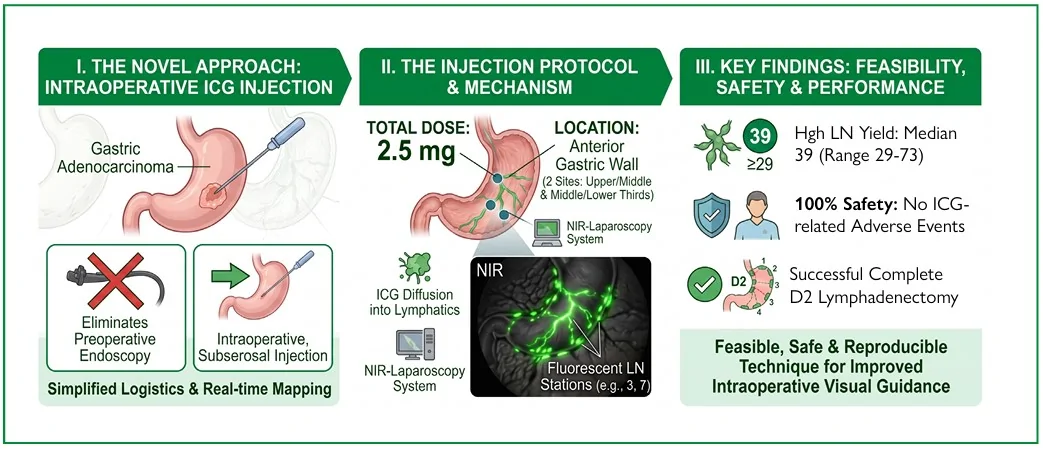

## RESUMO

**Racional:**

A linfadenectomia guiada por fluorescência com uso de verde de indocianina (ICG) melhora a visualização intraoperatória da drenagem linfática gástrica durante a gastrectomia. A maioria das técnicas relatadas baseia-se na injeção endoscópica pré-operatória, e os protocolos intraoperatórios padronizados são limitados.

**Objetivos:**

Avaliar a viabilidade, a segurança e a recuperação de linfonodos da injeção intraoperatória de ICG na subserosa para linfadenectomia D2 guiada por fluorescência durante a gastrectomia laparoscópica.

**Métodos:**

Foi realizado um estudo piloto descritivo e prospectivo em duas instituições. Foram incluídos pacientes consecutivos com adenocarcinoma gástrico submetidos a gastrectomia total ou subtotal laparoscópica com intenção curativa e linfadenectomia D2. O ICG foi injetado intraoperatoriamente na parede gástrica anterior (duas injeções de 1,25 mg) após exploração diagnóstica. Os desfechos incluíram viabilidade, segurança, distribuição da fluorescência pelas estações D2 e recuperação total de linfonodos.

**Resultados:**

Vinte pacientes foram incluídos. O protocolo foi implementado com sucesso em todos os casos, sem dificuldades técnicas nem eventos adversos relacionados ao ICG. A linfadenectomia D2 completa foi alcançada em 100% dos pacientes. A mediana do número de linfonodos recuperados foi de 39 (variação de 29 a 73), com =30 linfonodos em 95% dos casos. Linfonodos fluorescentes foram consistentemente identificados nas estações 3, 4, 6 e 7, com distribuição variável nas estações 5, 9, 8a, 11, 1 e 2, e ausentes na estação 12a.

**Conclusões:**

A injeção intraoperatória de ICG na camada subserosa é uma técnica viável, segura e reproduzível que permite a visualização consistente das principais bacias linfáticas gástricas sem a necessidade de injeção endoscópica pré-operatória.

## INTRODUCTION

 Gastric cancer continues to represent a significant burden worldwide and is the principal contributor to global cancer mortality^ 10,18, 32,34^. 

 Surgical resection with D2 lymphadenectomy continues to be the cornerstone of curative treatment^24,30^. However, the complex and variable vascular and lymphatic anatomy of the stomach makes lymph node dissection a technically demanding component of gastrectomy^ 12,13, 18,20, 35^. 

 Achieving an adequate lymphadenectomy is crucial not only for reducing residual disease, but also for ensuring accurate pathological staging, which directly influences prognosis and the indication for adjuvant therapy^23,26 ,28,37^. The quality of lymphadenectomy has long been recognized as a determinant of oncologic outcomes^11,16^. Although a minimum of 15 retrieved lymph nodes has historically been recommended for proper staging, recent studies indicate that harvesting 29 or more nodes provides superior staging accuracy and prognostic discrimination^1,4,28,29^. 

 Despite advances in minimally invasive techniques and high-definition visualization, identifying all relevant lymphatic basins and ensuring the completeness of D2 dissection remains challenging^9,13,21 ,36^. Incomplete retrieval of key nodal stations remains a concern, particularly in patients with complex anatomical variations or in centers with lower procedural volume^6,11,31^. 

 Fluorescence-guided surgery with indocyanine green (ICG) has gained attention as a tool to improve intraoperative visualization of lymphatic drainage pathways^2,7^. Because it allows real-time visualization of lymphatic flow and nodal stations under near-infrared imaging, this approach may improve the precision, reproducibility, and safety of lymphadenectomy^8,17,22^ . 

 However, agreement is lacking on the optimal timing, dosage, and route of ICG administration, and standardized protocols applicable across different surgical settings remain poorly defined^3,27,37^. Although a recent randomized trial has shown that intraoperative subserosal injection can achieve lymphatic mapping and oncologic outcomes comparable to those obtained with preoperative endoscopic submucosal injection, reproducible intraoperative injection strategies have not yet been clearly established^36^ . 

 In response to these gaps, we developed a new intraoperative ICG injection method designed to provide consistent and homogeneous fluorescence of the main gastric lymphatic basins without requiring preoperative endoscopic injection. 

 This study aims to describe the technical aspects of this protocol and to evaluate its feasibility and safety in patients with gastric cancer undergoing laparoscopic gastrectomy with curative intent. 

## METHODS

### Study design and patient selection

 This was a prospective descriptive pilot study conducted at two institutions: a University Hospital and an affiliated private tertiary center. Consecutive patients with a histologically confirmed diagnosis of gastric adenocarcinoma (clinical stage cT1-4a, N0-3, M0) who underwent D2 lymphadenectomy laparoscopic total or subtotal gastrectomy with curative intent were included. All procedures were performed from August 2018 to May 2021, during periods when laparoscopy towers equipped with fluorescence imaging technology were available as part of equipment demonstrations. 

 Exclusion criteria were age >80 years, known allergy to iodine or ICG, gastroesophageal junction tumors, previous gastric surgery, emergency surgery, and the need for multivisceral resections. All cases were reviewed by a multidisciplinary tumor board. Written informed consent for the use of intraoperative fluorescence imaging was obtained from each patient. The study was approved by the Institutional Ethics Committee (CEC-HCUCH-2025-014). 

 The primary aims of this study were to describe a novel intraoperative ICG injection protocol for fluorescence-guided D2 lymphadenectomy and to evaluate its feasibility, safety, and preliminary performance in terms of fluorescence distribution among lymph node stations and lymph node retrieval. 

### Fluorescence-guided lymphadenectomy protocol

#### Preparation of indocyanine green solution

 A vial containing 25 mg of ICG was reconstituted with 10 mL of sterile distilled water to obtain a 2.5 mg/mL solution. Two injections of 1.25 mg each were administered to achieve fluorescence of both first, and second, tier lymphatic barriers. Injection sites were selected according to the physiologic lymphatic drainage of the stomach described by Kinami et al.^15^ in the PTD classification, with the aim of obtaining consistent fluorescence across the lymph node stations included in a standard D2 lymphadenectomy. 

#### Intraoperative injection technique

 All injections were performed intraoperatively, immediately after diagnostic exploration and confirmation of resectability. Using a fine laparoscopic needle (Scalp Vein 21 Gauge), 0.5 mL of ICG solution was injected into the anterior gastric wall at each site, followed by 1 mL of sterile distilled water to ensure diffusion. The needle was then withdrawn approximately 1 cm, and an additional 1 mL of sterile distilled water was injected to flush the puncture tract. Finally, the needle was withdrawn by suction, avoiding dye extravasation into the operative field. 

 The two injection sites were located on the anterior surface of the stomach:

1. At the midpoint between the upper and middle thirds of the gastric body; and
2. At the midpoint between the middle and lower thirds.

The location of the injection points and the expected lymphatic diffusion pattern are illustrated in Figure 1. 

**Figure 1:**
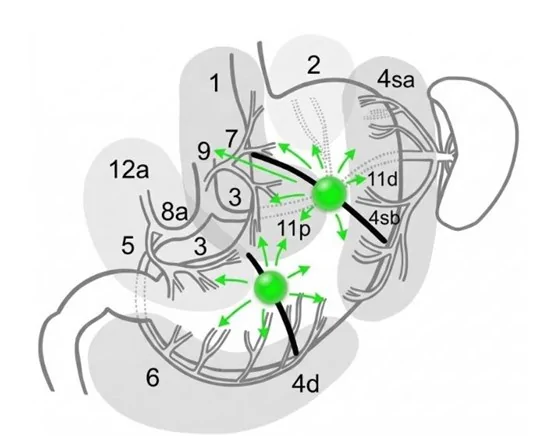
Schematic representation of the intraoperative indocyanine green injection protocol. Two injection sites were placed on the anterior gastric wall at the midpoints between the upper-middle and middle-lower thirds of the stomach. Green circles indicate injection sites, and arrows represent the expected lymphatic drainage toward D2 lymph node stations.

Source: personal archive

 This technique was designed to achieve homogeneous fluorescence across the five recognized lymphatic basins of the stomach, corresponding to the vascular pedicles supplying the greater and lesser curvatures. 

### Surgical technique

 All operations were performed laparoscopically under general anesthesia. The primary surgeon stood between the patient’s legs, assisted by one camera operator on the right and another on the left. Pneumoperitoneum was established to 15 mmHg using a Veress needle. Four 12-mm trocars were inserted (one supraum bilical, one in each flank for the surgeon’s hands, and one lateral for the assistant), together with a 5-mm port for liver retraction. 

 After exploration of the peritoneal cavity to exclude distant disease, the ICG injections were administered. Fluorescence imaging was conducted using one of three available near-infrared laparoscopic systems (Stryker 1588^®^, Stryker 1688^®^, or EleVision^®^). Total omentectomy was performed for tumors =T2 and limited for early lesions. The lymphadenectomy followed standard D2 dissection principles, removing stations 4sa, 4sb, 4d, and 6 along the gastroepiploic arcade; stations 5 and 12a along the right gastric and proper hepatic arteries; stations 3 and 1 by removing the gastrohepatic ligament and all lymphatic and fatty tissue up to the right paracardiac area; and stations 7, 8a, 9, and 11 along the left gastric, common hepatic, and splenic arteries. In total gastrectomy, paracardial station 2 was also included. 

 The duodenum was transected using a 60-mm linear stapler. In each lymph node station, the presence or absence of fluorescent lymph nodes was documented intraoperatively and later verified on recorded video footage. Reconstruction was performed in an antecolic Roux-en-Y fashion — either gastrojejunostomy or esophagojejunostomy — depending on the type of gastrectomy. The specimen was extracted through a Pfannenstiel incision. 

### Outcome measures

 The primary outcomes were:

- Feasibility of the technique (successful execution of planned D2 dissection).
- Safety (ICG-related adverse events).
- Fluorescence distribution across lymph node stations.
- Total number of retrieved lymph nodes.

Secondary outcomes included operative time, postoperative morbidity (Clavien-Dindo classification), length of hospital stay, and 90-day mortality. 

### Statistical analysis

 Given the exploratory nature of this study, only descriptive statistics were performed. 

 Continuous variables are reported as median and interquartile range or mean and standard deviation, as appropriate. Categorical variables are presented as frequencies and percentages. No comparative or inferential statistical analyses were performed, in accordance with the descriptive design of this preliminary study. 

## RESULTS

 A total of 20 consecutive patients undergoing laparoscopic gastrectomy with curative intent were included. The median age was 57.2±11.7 years, and 65% were male. Most patients presented with locally advanced gastric cancer (14/20, 70%), and total gastrectomy was performed in 18 cases (90%). The clinical characteristics of the patients are summarized in Table 1. 

**Table 1:** Clinical and manometric characteristics of the sample.

Variable		n=20
Mean age, years (SD)		57.2 (11.7)
Gender, n (%)		
	Men	13 (65)
	Women	7 (35)
	Mean body mass index, kg/m^2^ (SD)	26 (3.1)
ASA class, n (%)		
	I	0
	II	20 (100)
	III	0
Preoperative location, n (%)		
	Upper third	9 (45)
	Middle third	7 (35)
	Lower third	4 (20)
Comorbidities, n (%)		
	Hypertension	4 (20)
	Diabetes	3 (15)
	Hyperlipidemia	2 (10)
	Other	3 (15)
	Current or former smoker	11 (55)
	Alcohol consumption	9 (45)
Neoadjuvant chemotherapy, n (%)		
	FLOT	7 (35)
Adjuvant chemotherapy, n (%)		
	FLOT	5 (25)
	FOLFOX	9 (45)

SD: standard deviation; ASA: American Society of Anesthesiologists; FLOT: Fluorouracil, leucovorin, oxaliplatin and docetaxel; FOLFOX: 5-fluorouracil, oxaliplatin and leucovorin.

### Feasibility and safety of the intraoperative indocyanine green injection protocol

 The intraoperative ICG injection protocol was successfully implemented in all patients without technical difficulties. Only in three patients (15%) of the initial cases was there a slight spillage of ICG on the gastric surface, but this did not interfere with the subsequent evaluation of the presence or absence of fluorescent lymph nodes in the different lymph node groups. No ICG-related adverse events or allergic reactions were observed. Fluorescence imaging was feasible with all three near-infrared platforms used. A complete D2 lymphadenectomy, according to Japanese Gastric Cancer Association standards, was achieved in 100% of cases. 

 The median operative time was 282 minutes (interquartile range — IQR 160–410), and the median length of hospital stay was seven days (IQR 3–20). Three patients (15%) developed postoperative complications, all classified as Clavien Dindo grade II-IIIa, and there were no deaths within 90 days. Postoperative results are summarized in Table 2. 

**Table 2:** Operative and pathologic results.

Variable		n=20
Median operation time, minutes (min–max)		282 (160–410)
Extent of resection, n (%)		
	Distal gastrectomy	2 (10)
	Total gastrectomy	18 (90)
	R0 resection, n (%)	20 (100)
Extent of lymphadenectomy, n (%)		
	D1	0
	D1+	0
	D2	20 (100)
Median postoperative hospital stay, days (min–max)		7.0 (3–20)
Median of retrieved lymph nodes (min–max)		39 (29–73)
Benchmark 16 lymph nodes, n (%)		
	≥16 nodes	20 (100)
	<16 nodes	0
Benchmark 30 lymph nodes, n (%)		
	≥30 nodes	19 (95)
	<30 nodes	1 (5)
	Median of positive lymph nodes (min–max)	0 (0–11)
Histology, n (%)		
	Well or moderately differentiated	6 (30)
	Poorly differentiated	14 (70)
	Signet ring cell, n (%)	7 (35)
Pathologic T stage, n (%)		
	T1	6 (30)
	T2	3 (15)
	T3	8 (40)
	T4a	3 (15)
Pathologic N stage, n (%)		
	N0	11 (55)
	N1	4 (20)
	N2	2 (10)
	N3a	2 (10)
	N3b	1 (5)
Pathologic TNM stage (8th edition), n (%)		
	IA	5 (25)
	IB	2 (10)
	IIA	5 (25)
	IIB	4 (20)
	IIIA	1 (5)
	IIIB	2 (10)
	IIIC	1 (5)
	Total complications, n (%)	3 (15)
Type of complication, n (%)		
	Anastomotic leakage	2 (10)
	Intra-abdominal fluid collection	1 (5)
	Intra-abdominal bleeding	0
	Other	0
	90 days mortality, n (%)	0

SD: standard deviation; TNM: tumor, nodes and metastasis.

### Lymph node retrieval

 A total of 39 lymph nodes were retrieved per patient (median; range 29–73). All patients had at least 15 lymph nodes examined. In 19 of the 20 patients (95%), the number of retrieved lymph nodes was 29 or greater. Lymph node counts were recorded for all procedures. The distribution of lymph node retrieval remained within the reported range across both subtotal and total gastrectomies. 

### Fluorescence distribution across lymph node stations

 Fluorescence assessment was completed for all D2 lymph node stations in all patients. The number and proportion of stations exhibiting fluorescence are summarized in Figure 2. The initial lymphatic drainage pattern following injection is illustrated in Figure 3. Fluorescent lymph nodes were consistently identified in 100% of stations 3, 4, 6, and 7, as demonstrated in representative high-frequency stations (Figure 4). In contrast, fluorescence was observed less frequently in stations 5, 9, 8a, 11, 1, and 2, particularly in more central nodal basins (Figure 5). No fluorescent lymph nodes were identified in station 12a. 

**Figure 2:**
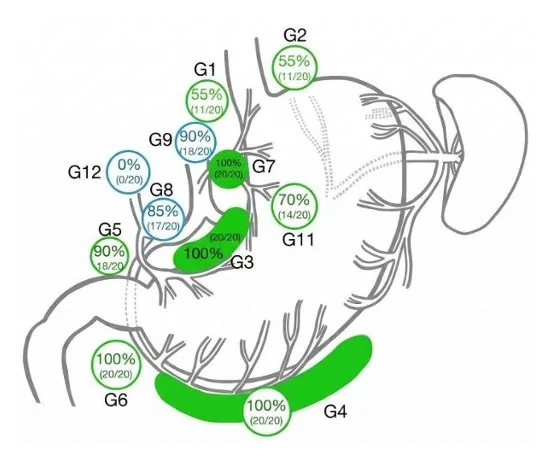
Distribution of fluorescence across D2 lymph node stations. The proportion of patients demonstrating fluorescent lymph nodes in each station is shown as percentage and absolute count (n/20). Uniform fluorescence was observed in stations 3, 4, 6, and 7, whereas variable fluorescence was detected in stations 5, 8a, 9, 11, 1, and 2. No fluorescence was observed in station 12a.

Source: personal archive

**Figure 3:**
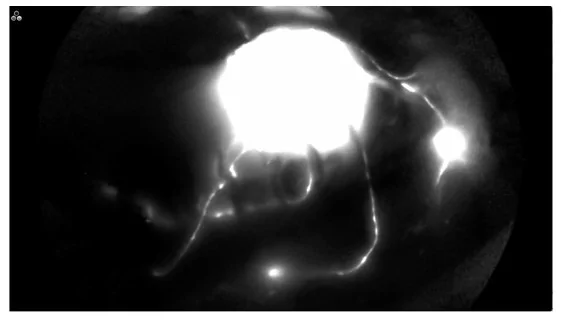
Lymphatic mapping from injection site. Inferior injection demonstrating lymphatic drainage pathways toward the greater curvature, visualized in contrast mode.

Source: Personal archive.

**Figure 4:**
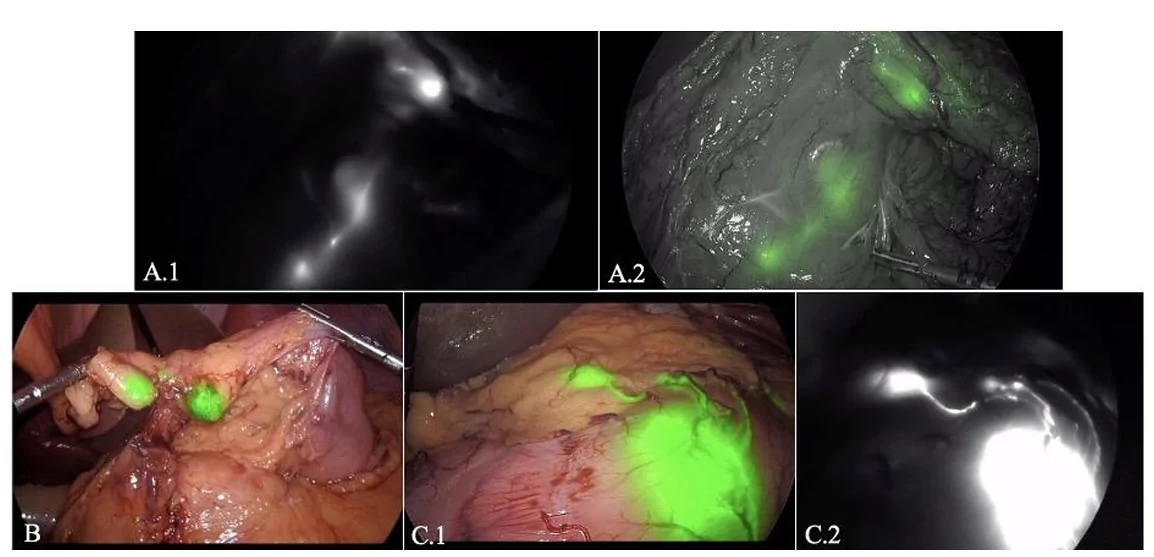
High-frequency fluorescent lymph node stations (stations 3, 4, and 6). A. Distal station 4 (upper left) and infrapyloric station 6 (lower right), showing lymphatic channels and nodes in relation to the origin of the right gastroepiploic vessels, visualized using contrast (grayscale) and near-infrared fluorescence (ENV) modes. B. Infrapyloric station 6 dissection demonstrating fluorescent lymph nodes directed toward the surgical specimen. The right gastroepiploic vessels have been divided, with the distal dissection margin at the duodenum. Visualized using SPY overlay (white light + fluorescence). C. Station 3 (hepatogastric ligament), visualized using SPY overlay and contrast modes.

Source: Personal archive.

**Figure 5:**
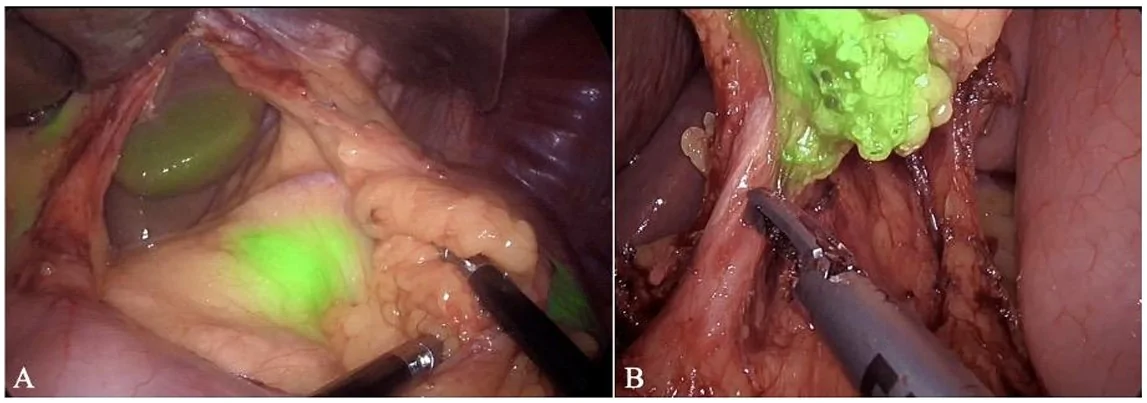
Lower-frequency fluorescence in central nodal stations (stations 8 and 9). A. Stations 8a and 9 (common hepatic artery and celiac trunk), prior to dissection, visualized using SPY overlay mode. B. Left gastric artery with surrounding lymphatic and fatty tissue directed toward the surgical specimen, visualized using SPY overlay mode.

Source: Personal archive.

### Specimen handling

 All specimens were extracted through a Pfannenstiel incision. No intraoperative specimen fragmentation or margin compromise was reported. Macroscopic evaluation confirmed en bloc resection in all cases. 

## DISCUSSION

 This study describes the development and early application of an intraoperative ICG injection protocol intended to support fluorescence-guided D2 lymphadenectomy during laparoscopic gastrectomy. In this consecutive series, the method was carried out without technical difficulties, allowed real-time visualization of the main gastric lymphatic pathways, and permitted a systematic evaluation of fluorescence in all D2 lymph node stations. No adverse reactions to ICG were recorded, and a standard D2 lymphadenectomy was completed in every patient. The technical details outlined here provide a reproducible framework that may be refined and assessed further in larger cohorts. 

### Current evidence and contextualization of the technique

 Fluorescence-guided lymphadenectomy in gastric cancer has been predominantly explored through preoperative endoscopic submucosal injection of ICG^5,33^. This approach forms the basis of most contemporary studies evaluating sentinel node navigation surgery and fluorescence-assisted D2 dissection^25,33^. Protocols commonly involve preoperative four-quadrant peritumoral injections of ICG, typically administered either the day before surgery or a few hours prior to the procedure^14^. This approach has been associated with improved identification of lymphatic basins and enhanced completeness of D2 lymphadenectomy in comparative series^2,17^. However, these techniques require access to specialized endoscopic equipment and personnel, involve additional procedural coordination, and are subject to variability in injection timing, diffusion kinetics, and submucosal plane accuracy. As a result, their implementation outside high-volume centers remains inconsistent^25^, and interest has grown in developing simplified intraoperative methods that avoid these logistical challenges. 

 Beyond differences in injection modality, current literature exhibits substantial heterogeneity in dosing, concentration, number of injection points, and timing of administration^27,37^. Reported total doses range widely, and studies differ in whether injections are submucosal, subserosal, or mixed, making it difficult to compare fluorescence distribution or to standardize best practices^27^. Recent data showed that intraoperative subserosal injection achieves lymphatic mapping and nodal retrieval comparable to preoperative endoscopic submucosal injection^36^. These findings suggest that an intraoperative approach may represent a simpler alternative without compromising oncologic adequacy. Across studies, fluorescence guidance has been associated with higher lymph node yields and lower rates of D2 non-compliance. Some randomized data also suggest potential improvements in disease-free and overall survival in patients undergoing fluorescence-assisted gastrectomy^8,17,19^. 

 The fluorescence patterns observed in the present study align with anatomical expectations described in lymphatic mapping literature. Stations along the lesser curvature, greater curvature, and left gastric artery demonstrated uniformly high fluorescence, whereas proximal stations and the hepatoduodenal ligament showed variable or absent uptake, consistent with their more complex or limited anterior drainage routes. These findings reflect the influence of lymphatic anatomy on tracer behavior and highlight the need for further research to determine how fluorescence in specific stations correlates with pathological nodal involvement and clinical outcomes. 

### Technical findings and procedural performance

 Postoperative morbidity was low despite the cohort being predominantly composed of patients with locally advanced tumors. The intraoperative ICG protocol yielded a clear and reproducible fluorescence pattern across the principal lymphatic territories of the stomach. Uniform fluorescence was observed in stations located along the lesser and greater curvatures and along the left gastric and gastroepiploic vascular arcades, including the infra-pyloric group (G6), which is anatomically more challenging in terms of performing a proper dissection, reflecting consistent tracer diffusion toward nodes typically included in a standard D2 dissection. Fluorescence was also detected in a high proportion of stations located along the common hepatic artery and celiac axis, allowing a comprehensive intraoperative mapping of these areas. In contrast, fluorescence was less frequently observed in proximal paracardial stations and was absent in station 12a, highlighting heterogeneous uptake patterns in lymphatic regions with more variable or limited drainage from the anterior gastric wall. 

 The protocol allowed a complete assessment of fluorescence in all stations in every case, and documentation was feasible both intraoperatively and through postoperative video review. The standardized dosing, injection sites, and injection sequence facilitated consistent execution across procedures. The technique integrated seamlessly into the operative workflow without prolonging the procedure or requiring additional personnel or endoscopic equipment. Overall, these technical features support the reproducibility of the protocol and its potential applicability in varied surgical settings. 

### Potential clinical implications

 The fluorescence patterns observed may assist intraoperative decision-making during gastrectomy. Real-time fluorescence imaging may facilitate a more systematic approach to D2 lymphadenectomy by delineating lymphatic basins that are not always easily identifiable under conventional whitelight imaging. The technique may also help verify the completeness of dissection within predefined anatomical boundaries, particularly in areas where lymphatic planes can be obscured or distorted, effectively providing a real-time “quality control” of the resection by confirming that fluorescent lymph nodes are adequately removed. Additionally, this approach may support surgical training by enhancing anatomical understanding and potentially facilitating the learning curve for surgeons and teams adopting minimally invasive techniques for gastric cancer. 

 Because the protocol does not require preoperative endoscopic injection, it may offer logistical advantages in settings where endoscopic resources are limited or where scheduling constraints hinder coordinated preoperative mapping. The ability to perform the entire process intraoperatively may also enhance workflow efficiency and reduce variability associated with timing, diffusion kinetics, or injection technique. The reproducibility observed in this initial series indicates that the protocol could serve as a practical adjunct to standard lymphadenectomy and support efforts to standardize fluorescenceguided approaches in centers performing minimally invasive gastric cancer surgery. 

### Limitations

 This study has several limitations that should be considered when interpreting its findings. The sample size was small and derived from a single surgical team, which restricts the generalizability of the results. The study was designed as a descriptive pilot evaluation without a control group, and therefore no comparative conclusions regarding the oncologic performance or clinical benefit of the protocol can be drawn. The predominance of total gastrectomies, advanced tumors and preoperative chemotherapy within the cohort may influence the observed patterns of lymph node retrieval and fluorescence distribution. 

 Fluorescence assessment was based on visual inspection under near-infrared imaging, and no correlation was performed between fluorescence and pathological nodal status. Additionally, the study evaluated only one dosing strategy and a single set of injection sites; alternative injection locations or volumes were not explored and may yield different fluorescence patterns. Finally, the use of different near-infrared platforms may introduce variability in fluorescence detection, although all systems allow adequate visualization in this series. 

## CONCLUSIONS

 This study describes a novel intraoperative ICG injection protocol for fluorescence-guided D2 lymphadenectomy during laparoscopic gastrectomy, and demonstrates its feasibility, safety, and reproducibility in a consecutive cohort. The protocol enabled consistent real-time visualization of major gastric lymphatic basins, and allowed a systematic assessment of fluorescence across all D2 lymph node stations without requiring preoperative endoscopic injection. These preliminary findings support the technical applicability of this approach and should be further evaluated in larger series aimed at validating its clinical utility and exploring its potential role within standardized fluorescence-guided lymphadenectomy workflows. 

## Data Availability

The datasets generated and/or analyzed during the current study are available from the corresponding author upon reasonable request.

## References

[1] Amin MB, Greene FL, Edge SB, Compton CC, Gershenwald JE, Brookland RK (2017). The Eighth Edition AJCC Cancer Staging Manual: Continuing to build a bridge from a population-based to a more “personalized” approach to cancer staging. CA Cancer J Clin.

[2] Baiocchi GL, Molfino S, Molteni B, Quarti L, Arcangeli G, Manenti S (2020). Fluorescence-guided lymphadenectomy in gastric cancer: a prospective western series. Updates Surg.

[3] Belia F, Biondi A, Agnes A, Santocchi P, Laurino A, Lorenzon L (2022). The Use of Indocyanine Green (ICG) and NearInfrared (NIR) fluorescence-guided imaging in gastric cancer surgery: a narrative review. Front Surg.

[4] Biffi R, Botteri E, Cenciarelli S, Luca F, Pozzi S, Valvo M (2011). Impact on survival of the number of lymph nodes removed in patients with node-negative gastric cancer submitted to extended lymph node dissection. Eur J Surg Oncol.

[5] Calcara C, Cocciolillo S, Canavesio YM, Adamo V, Carenzi S, Lucci DI (2023). Endoscopic fluorescent lymphography for gastric cancer. World J Gastrointest Endosc.

[6] Eshuis WJ, van Berge Henegouwen MI, Draaisma WA, Gisbertz SS (2018). Compliance to D2 lymphadenectomy in laparoscopic gastrectomy. Updates Surg.

[7] He M, Jiang Z, Wang C, Hao Z, An J, Shen J (2018). Diagnostic value of near-infrared or fluorescent indocyanine green-guided sentinel lymph node mapping in gastric cancer: a systematic review and meta-analysis. J Surg Oncol.

[8] Huang ZN, Tang YH, Zhong Q, Li P, Xie JW, Wang JB (2024). Assessment of laparoscopic indocyanine green tracer-guided lymphadenectomy after neoadjuvant chemotherapy for locally advanced gastric cancer: a randomized controlled trial. Ann Surg.

[9] Hyung WJ, Yang HK, Park YK, Lee HJ, An JY, Kim W (2020). Long-term outcomes of laparoscopic distal gastrectomy for locally advanced gastric cancer: the KLASS-02-RCT Randomized Clinical Trial. J Clin Oncol.

[10] Ilic M, Ilic I (2022). Epidemiology of stomach cancer. World J Gastroenterol.

[11] Degiuli M, Sasako M, Ponti A, Vendrame A, Tomatis M, Mazza C (2014). Randomized clinical trial comparing survival after D1 or D2 gastrectomy for gastric cancer. Br J Surg.

[12] Japanese Gastric Cancer Association (2011). Japanese classification of gastric carcinoma: 3rd English edition. Gastric Cancer.

[13] Japanese Gastric Cancer Association (2017). Japanese gastric cancer treatment guidelines 2014 (ver. 4). Gastric Cancer.

[14] Kelder W, Nimura H, Takahashi N, Mitsumori N, van Dam GM, Yanaga K (2010). Sentinel node mapping with indocyanine green (ICG) and infrared ray detection in early gastric cancer: an accurate method that enables a limited lymphadenectomy. Eur J Surg Oncol.

[15] Kinami S, Fujimura T, Ojima E, Fushida S, Ojima T, Funaki H (2008). PTD classification: proposal for a new classification of gastric cancer location based on physiological lymphatic flow. Int J Clin Oncol.

[16] Kung CH, Tsai JA, Lundell L, Johansson J, Nilsson M, Lindblad M (2020). Nationwide study of the impact of D2 lymphadenectomy on survival after gastric cancer surgery. BJS Open.

[17] Kwon IG, Son T, Kim HI, Hyung WJ (2019). Fluorescent lymphography-guided lymphadenectomy during robotic radical gastrectomy for gastric cancer. JAMA Surg.

[18] Lirosi MC, Biondi A, Ricci R (2017). Surgical anatomy of gastric lymphatic drainage. Transl Gastroenterol Hepatol.

[19] Luo G, Zhang Y, Guo P, Wang L, Huang Y, Li K (2017). Global patterns and trends in stomach cancer incidence: age, period and birth cohort analysis. Int J Cancer.

[20] Maruyama K, Gunvén P, Okabayashi K, Sasako M, Kinoshita T (1989). Lymph node metastases of gastric cancer: general pattern in 1931 patients. Ann Surg.

[21] Norero E, Ceroni M, Martinez C, Muñoz R, Mejia R, Morales E (2024). Long-term survival after laparoscopic total gastrectomy for early and advanced gastric cancer. Single center experience in 100 cases. ABCD Arq Bras Cir Dig.

[22] Park JY, Kim YW, Ryu KW, Nam BH, Lee YJ, Jeong SH (2016). Assessment of laparoscopic stomach preserving surgery with sentinel basin dissection versus standard gastrectomy with lymphadenectomy in early gastric cancer-A multicenter randomized phase III clinical trial (SENORITA trial) protocol. BMC Cancer.

[23] Ramos MFKP, Pereira MA, Albuquerque AF, Viana EF, Costa WL, Sanches SRA (2025). Implementation of the recommendations of the II Brazilian Consensus On Gastric Cancer in clinical practice: a multicenter study of the Brazilian Gastric Cancer Association. Arq Bras Cir Dig.

[24] Sasako M, Sano T, Yamamoto S, Kurokawa Y, Nashimoto A, Kurita A (2008). D2 lymphadenectomy alone or with para-aortic nodal dissection for gastric cancer. N Engl J Med.

[25] Sherwinter DA, Boni L, Bouvet M, Ferri L, Hyung WJ, Ishizawa T (2022). Use of fluorescence imaging and indocyanine green for sentinel node mapping during gastric cancer surgery: results of an intercontinental Delphi survey. Surgery.

[26] Siewert JR, Böttcher K, Stein HJ, Roder JD (1998). Relevant prognostic factors in gastric cancer: ten-year results of the German Gastric Cancer Study. Ann Surg.

[27] Slim N, Anbu D, Darzi A, Elson DS, Peters CJ (2025). The use of indocyanine green and near-infrared fluorescence in the detection of metastatic lymph nodes during oesophageal and gastric cancer resection: a systematic review and meta analysis. Surg Endosc.

[28] Smith DD, Schwarz RR, Schwarz RE (2005). Impact of total lymph node count on staging and survival after gastrectomy for gastric cancer: data from a large US-population database. J Clin Oncol.

[29] Son T, Hyung WJ, Lee JH, Kim YM, Kim HI, An JY (2012). Clinical implication of an insufficient number of examined lymph nodes after curative resection for gastric cancer. Cancer.

[30] Songun I, Putter H, Kranenbarg EMK, Sasako M, van de Velde CJH (2010). Surgical treatment of gastric cancer: 15-year follow-up results of the randomised nationwide Dutch D1D2 trial. Lancet Oncol.

[31] Strong VE, Song KY, Park CH, Jacks LM, Gonen M, Shah M (2010). Comparison of gastric cancer survival following R0 resection in the United States and Korea using an internationally validated nomogram. Ann Surg.

[32] Sung H, Ferlay J, Siegel RL, Laversanne M, Soerjomataram I, Jemal A (2021). Global cancer statistics 2020: GLOBOCAN estimates of incidence and mortality worldwide for 36 cancers in 185 countries. CA Cancer J Clin.

[33] Tajima Y, Yamazaki K, Masuda Y, Kato M, Yasuda D, Aoki T (2009). Sentinel node mapping guided by indocyanine green fluorescence imaging in gastric cancer. Ann Surg.

[34] Thrift AP, El-Serag HB (2020). Burden of gastric cancer. Clin Gastroenterol Hepatol.

[35] Wei J, Bu Z (2020). Sentinel lymph node detection for gastric cancer: promise or pitfall?. Surg Oncol.

[36] Zhong Q, Shang-Guan ZX, Liu ZY, Wu D, Huang ZN, Wang HG (2025). Comparison of a submucosal and subserosal approach in ICG-guided laparoscopic lymphadenectomy in gastric cancer patients: long-term outcomes of a phase 3 randomized clinical trial. Int J Surg.

[37] Zhuang C, Yang HK (2025). Fluorescence guided surgery in gastric cancer: what do we have and what can we do?. Ann Gastroenterol Surg.

